# Median Estimation with Quantile Transformations: Applications to Stratified Two-Phase Sampling

**DOI:** 10.3390/e27121191

**Published:** 2025-11-24

**Authors:** Fatimah A. Almulhim, Hassan M. Aljohani

**Affiliations:** 1 Department of Mathematical Sciences, College of Science, Princess Nourah bint Abdulrahman University, P.O. Box 84428, Riyadh 11671, Saudi Arabia; faalmulhim@pnu.edu.sa; 2 Department of Mathematics and Statistics, College of Science, Taif University, P.O. Box 11099, Taif 21944, Saudi Arabia

**Keywords:** median estimation, auxiliary information, quantile transformations, stratified two-phase sampling, Monte Carlo simulation, bias, mean squared errors, relative efficiency, 62D05

## Abstract

Most traditional estimators assume normality and remain sensitive to extreme observations, which limits their usefulness in practical applications. To improve accuracy, we introduce quintile-based median estimators using transformation methods in a stratified two-phase sampling technique. The design allows for efficient use of auxiliary data and enhances robustness across heterogeneous strata. Stratified sampling further reduces variability by ensuring representation from all subgroups within the population. Bias and mean squared error expressions are obtained through first-order approximations. The efficiency of the proposed estimators is evaluated using the mean squared error (MSE) as the benchmark criterion. The effectiveness of the proposed estimators is examined by conducting simulations under various skewed distributions. To strengthen the conclusions, additional analysis is performed on real population datasets. Simulation and empirical studies confirm the superior performance of the proposed methods. The findings show that the suggested estimators perform well in practical situations involving median estimation as well as achieving higher precision and effectiveness than existing estimators.

## 1. Introduction

In survey research, obtaining precise estimates is more challenging when the population is biased or includes outliers. The median provides a more accurate picture of central tendency in such cases, while the arithmetic mean can provide misleading findings. In different fields such as monitoring computer network traffic, ecological species distribution studies, and public policy evaluations, where extreme responses significantly influence average outcomes, median-based analysis has achieved significance due to its statistical reliability. Stratified two-phase sampling is an effective modification of stratification methods that achieves a balance between survey efficiency and precision. The population is divided into strata, from which the initial sample is selected for each group, followed by a second subsample to enhance estimations. This method effectively estimates the finite population median due to its robustness against outliers and skewness. Applications in real life highlight the method’s improvements: classifying by occupation in economic surveys helps in the production of accurate estimates of median income, and stratifying by age or illness severity in medical studies improves the computation of median recovery times. These applications emphasize the effectiveness of stratified two-phase sampling as an accurate method of survey study. Details concerning auxiliary information are available in [1,2,3,4].

The precision of median estimation has progressively improved through the use of auxiliary information. Foundational studies [5,6,7] introduced the role of auxiliary variables in developing efficient estimators. Regression and ratio-based approaches [8,9] further enhanced estimation accuracy. Later, generalized estimators employing multiple auxiliary variables and double sampling methods [10] were proposed to address the absence of complete auxiliary data [11]. Two-phase designs offered an optimal balance between cost and precision [12], while known auxiliary medians enabled the formulation of unbiased estimators [13]. An efficient class of ratio-cum-median estimators for estimating the population median was proposed by [14], while [15] discussed the formulation of efficiency for median estimation under a fixed cost in survey sampling. A detailed understanding of robust and exploratory data analysis was introduced by [16], providing valuable insights into handling outliers and non-normal data structures. This foundation supports the development of more reliable and efficient statistical estimators in complex sampling scenarios.

Over time, scholars have also explored transformation-based and non-parametric strategies to enhance the robustness of median estimators under non-normal or skewed conditions. The inclusion of auxiliary information not only improves efficiency but also mitigates the influence of extreme values, ensuring stability across diverse population structures. Recent advancements integrate techniques such as exponential-type transformations, calibration adjustments, and Bayesian refinements to achieve higher precision without inflating survey costs. Exponential-Poisson parameters estimation in moving extremes ranked set sampling design discussed by [17]. Some generalized classes of estimators under two-phase sampling approach introduced by [18,19]. Improved median estimation in stratified surveys via non traditional auxiliary measures discussed by [20,21]. Furthermore, simulation-based validation and empirical analyses using real-world datasets have confirmed the practical utility of these enhanced estimators in socioeconomic, agricultural, and industrial sampling frameworks. The detailed reviews on this topic can be found in [22,23,24,25,26,27,28,29,30], which collectively provide comprehensive insights into the theoretical evolution, comparative efficiency, and modern extensions of median estimation methodologies.

Populations that deviate from normality or display skewed patterns often challenge the reliability of conventional estimators such as the ratio, regression, exponential, and product types. These estimators assume data symmetry and tend to perform poorly in the presence of extreme or irregular values. To overcome the constraints identified by [28], this study introduces a class of transformation-based estimators constructed within a two-phase stratified random sampling design. The proposed framework strengthens estimator performance by combining the robustness of the central measure with the efficiency improvement obtained through auxiliary data collected during the first phase. This dual advantage makes our estimators uniquely suited for modern survey contexts, where complex populations rarely align with standard distributions. These transformations capture distributional features such as spread, skewness, and tail behavior, allowing the proposed estimators to remain efficient and stable even in highly irregular settings. This technique is especially effective for heterogeneous and unstable datasets, such as those produced by climate-sensitive agricultural yields, defective manufacturing processes, or uneven academic performance distributions. The proposed estimator class offers major improvements in survey methodology. It performs reliably in skewed or non-normal populations and remains stable against outliers, ensuring accurate results under real data conditions. The two-phase stratified design provides cost-efficient robustness by utilizing first-phase auxiliary data, while transformation-based refinements enhance efficiency beyond classical ratio and regression estimators. Its flexibility supports applications in agriculture, healthcare, and economics, with simulation results confirming strong cross-distributional consistency and reliability.

### 1.1. Quantile Background

Quantiles present a distributional summary by dividing the data into ordered sets and identifying the number that represents such divisions. The *p*th quantile of a continuous random variable *X*, with distribution function F(x), is defined as

Q(p)=inf{x:F(x)≥p},0<p<1.

The median Q(0.50), the first and third quartiles Q(0.25) and Q(0.75), and quantile-based measures like the interquartile range and median absolute deviation are significant special scenarios. Unlike means and variances, quantiles are not affected by extreme values and are also stable in case of skewed or heavy-tailed distributions. Due to such attributes, quantile-based methods are particularly well adapted to robust inference, especially in survey sampling situations where population values often contain strong outliers or are no longer normality.

### 1.2. Research Objectives

The primary purpose of this research is to develop efficient median estimators when it comes to stratified two-phase sampling and in particular populations that have skewness or extreme cases. The proposed approach increases accuracy and robustness through quantile-based modifications in order to effectively model distributional characteristics such as shape, spread, and tail behavior. The paper also aims at obtaining theoretical features of these estimators, including expressions of mean squared error and first-order bias and constructing analytic. Extensive Monte Carlo simulations are carried out over several skewed distributions to evaluate practical performance, and empirical utility is demonstrated by analyzing actual survey data from various application domains.

### 1.3. Key Contributions

A general family of double-exponential median estimators using quantile-based measures, such as quartile deviation, trimmed mean, decile mean, quartile average, product measure, interquartile range, and median absolute deviation, is shown in this study. The suggested estimators overcome the drawbacks of existing two-phase stratified median techniques by improving robustness to skewness, wide tails, and heterogeneous strata. Under the stratified two-phase approach, unified theoretical results for bias and mean squared error are presented, together with analytical superiority criteria that illustrate situations in which new estimators attain higher efficiency. In comparison to conventional and modern estimators, empirical assessments, involving simulations and actual datasets, consistently show improvements in mean squared error. With every aspect considered, the study provides a flexible methodological framework that can be expanded to include multi-phase sampling designs and additional reliable location measures.

## 2. Methodology and Notations

In this section, we begin by laying out the notations and earlier work on median estimation. Assume a population of size *N* is expressed as:

$$\xi =\{\xi_{1},\xi_{2},\ldots ,\xi_{N}\}.$$

Let the population be separated into *L* strata without overlap, with the *h*th stratum including $N_{h}$ units, such that

$$\sum_{h=1}^{L}N_{h}=N.$$

For every stratum, *Y* is the main variable under study, while *X* is treated as an auxiliary input to provide support for estimation. The first phase consists of selecting a simple random sample without replacement of size $m_{h}$ from stratum *h*th, where the overall sample size is given by:

$$\sum_{h=1}^{L}m_{h}=m.$$

Only values of the auxiliary variable *x* are collected at this point. This phase helps in estimating the overall population median of *X* denoted by $M_{x}$.

The second-phase sample sizes in each stratum, drawn from the corresponding first-phase samples, are denoted by $n_{h}$ with $n_{h}<m_{h}$, satisfy the condition

$$\sum_{h=1}^{L}n_{h}=n.$$

All sub-sampling is carried out using simple random sampling without replacement (SRSWOR) within each stratum. This design ensures that auxiliary information obtained in the first phase enhances estimation in the second phase. In this stage, both *Y* and *X* values are recorded.

In the *h*th stratum, the medians of *Y* and *X* are denoted by $M_{yh}$ and $M_{xh}$. Their two-phase sample analogues are ${\overset{´}{M}}_{x_{h}},{\hat{M}}_{x_{h}},$ and ${\hat{M}}_{yh}$. The probability densities at the respective medians are expressed as $f_{yh}\left(M_{yh}\right)$ and $f_{xh}\left(M_{xh}\right)$. The correlation between these medians is defined by

$$\rho \left(M_{yh},M_{xh}\right)=4P_{11h}\left(y_{h},x_{h}\right)-1,$$

with $P_{11h}$ representing the probability that

$$P(y_{h}\leq M_{yh},\;x_{h}\leq M_{xh}).$$

The following expressions for relative error and corresponding expected values serve as the basis for first-order approximations of biases and mean squared errors:

$$e_{0h}=\left(\frac{{\hat{M}}_{yh}-M_{yh}}{M_{yh}}\right),$$

$$e_{1h}=\left(\frac{{\hat{M}}_{xh}-M_{xh}}{M_{xh}}\right)$$

and

$$e_{2h}=\left(\frac{{\overset{´}{M}}_{xh}-M_{xh}}{M_{xh}}\right),$$

such that $E\left(e_{ih}\right)=0$ for i=0,1,2.

Moreover,

$$E\left(e_{0h}^{2}\right)=\theta_{1h}C_{M_{yh}}^{2},$$

$$E\left(e_{1h}^{2}\right)=\theta_{1h}C_{M_{xh}}^{2},$$

$$E\left(e_{2h}^{2}\right)=\theta_{2h}C_{M_{xh}}^{2},$$

$$E\left(e_{0h}e_{1h}\right)=\theta_{1h}C_{M_{yxh}}=\rho_{yxh}C_{M_{yh}}C_{M_{xh}},$$

$$E\left(e_{0h}e_{2h}\right)=\theta_{2h}C_{M_{yxh}}=\rho_{yxh}C_{M_{yh}}C_{M_{xh}},$$

$$E\left(e_{1h}e_{2h}\right)=\theta_{2h}C_{M_{xh}}^{2},$$

where

$$C_{M_{yh}}=\frac{1}{M_{yh}f_{yh}\left(M_{yh}\right)},$$

$$C_{M_{xh}}=\frac{1}{M_{yh}f_{xh}\left(M_{xh}\right)},$$

$$\mathrm{Cov}\left(M_{y_{h}},M_{x_{h}}\right)=\frac{\rho_{yxh}}{4f_{yh}\left(M_{yh}\right)f_{xh}\left(M_{xh}\right)},$$

$$\theta_{1h}=\frac{1}{4}\left(\frac{1}{n_{h}}-\frac{1}{N_{h}}\right),\;\theta_{2h}=\frac{1}{4}\left(\frac{1}{m_{h}}-\frac{1}{N_{h}}\right),\;\;\theta_{3h}=\frac{1}{4}\left(\frac{1}{n_{h}}-\frac{1}{m_{h}}\right).$$

Table 1 summarizes all the notations and symbols used in this study.

## 3. Stratified Two-Phase Existing Estimators

Various researchers have introduced a number of median estimators which are characterized by different degree of efficiency with respect to the sampling design, and the underlying population. We present the summarization of these estimators along with their theoretical properties, including bias, variance, and mean squared error, in this section. The review not only offers a point of reference to compare but also shows the possible ineffectiveness of current methods, which inspires the necessity of more effective ones.

The conventional sample median estimator and its variance expression, originally introduced by [5], are extended to the framework of stratified two-phase sampling and are defined as follows:

(1) $${\hat{M}}_{yst}=\sum_{h=1}^{L}W_{h}{\hat{M}}_{y_{h}}$$

and

(2) $$Var\left({\hat{M}}_{yst}\right)=\sum_{h=1}^{L}\theta_{1h}W_{h}^{2}M_{yh}^{2}C_{M_{yh}}^{2}.$$

The ratio estimator proposed by [10] in the context of two-phase sampling is adapted in this study to stratified two-phase sampling, defined as:

(3) $${\hat{M}}_{Ast}=\sum_{h=1}^{L}W_{h}\left(\frac{{\hat{M}}_{yh}}{{\hat{M}}_{xh}}\right){\overset{´}{M}}_{xh}.$$

The following expressions represent the first-order approximations of the bias and MSE for ${\hat{M}}_{Ast}$:

(4) $$Bias\left({\hat{M}}_{Ast}\right)≅\sum_{h=1}^{L}\theta_{3h}W_{h}M_{yh}\left(C_{M_{xh}}^{2}-C_{M_{yxh}}\right)$$

and

(5) $$MSE\left({\hat{M}}_{Ast}\right)≅\sum_{h=1}^{L}W_{h}^{2}M_{yh}^{2}\left[\theta_{1h}C_{M_{yh}}^{2}+\theta_{3h}\left(C_{M_{xh}}^{2}-2C_{M_{yxh}}\right)\right].$$

In line with the approach described in [13], the difference-type estimator is modified for stratified two-phase sampling, leading to the expression for ${\hat{M}}_{D_{1st}}$:

(6) $${\hat{M}}_{D_{1st}}=\sum_{h=1}^{L}W_{h}\left[{\hat{M}}_{yh}+d_{1h}\left({\overset{´}{M}}_{xh}-{\hat{M}}_{xh}\right)\right].$$

Based on the first-order approximation, the minimum MSE of ${\hat{M}}_{D_{1}}$ corresponding to the optimal $d_{1h}$ is defined as:

(7) $$MSE{\left({\hat{M}}_{D_{1st}}\right)}_{min}≅\sum_{h=1}^{L}W_{h}^{2}M_{yh}^{2}C_{M_{yh}}^{2}\left(\theta_{1h}-\theta_{3h}\rho_{yxh}^{2}\right),$$

where

$$d_{1h\left(opt\right)}=\frac{\rho_{yxh}M_{yh}C_{M_{yh}}}{M_{xh}C_{M_{xh}}}.$$

Within the two-phase sampling framework, Ref. [31] introduced median versions of the exponential ratio and product estimators. In this study, these estimators are further generalized to stratified two-phase sampling as follows:

(8) $${\hat{M}}_{Rest}=\sum_{h=1}^{L}W_{h}{\hat{M}}_{yh}\exp\left(\frac{{\overset{´}{M}}_{xh}-{\hat{M}}_{xh}}{{\overset{´}{M}}_{xh}+{\hat{M}}_{xh}}\right)$$

and

(9) $${\hat{M}}_{Pest}=\sum_{h=1}^{L}W_{h}{\hat{M}}_{yh}\exp\left(\frac{{\hat{M}}_{xh}-{\overset{´}{M}}_{xh}}{{\overset{´}{M}}_{xh}+{\hat{M}}_{xh}}\right).$$

The first-order approximations of the biases and MSEs for (${\hat{M}}_{R_{est}},{\hat{M}}_{P_{est}}$) are given below:

(10) $$Bias\left({\hat{M}}_{Rest}\right)≅\frac{1}{2}\sum_{h=1}^{L}\theta_{3h}W_{h}M_{yh}\left(\frac{3}{4}C_{M_{xh}}^{2}-C_{M_{yxh}}\right),$$

(11) $$Bias\left({\hat{M}}_{Pest}\right)≅\frac{1}{2}\sum_{h=1}^{L}\theta_{3h}W_{h}M_{y}\left(\frac{3}{4}C_{M_{x}}^{2}+C_{M_{yx}}\right),$$

(12) $$MSE\left({\hat{M}}_{Rest}\right)≅\sum_{h=1}^{L}W_{h}^{2}M_{yh}^{2}\left[\theta_{1h}C_{M_{yh}}^{2}+\theta_{3h}C_{M_{xh}}^{2}\left(\frac{1}{4}-K_{h}\right)\right]$$

and

(13) $$MSE\left({\hat{M}}_{Pest}\right)≅\sum_{h=1}^{L}W_{h}^{2}M_{yh}^{2}\left[\theta_{1h}C_{M_{yh}}^{2}+\theta_{3h}C_{M_{xh}}^{2}\left(\frac{1}{4}+K_{h}\right)\right],$$

where

$$K_{h}=\frac{\rho_{yxh}C_{M_{yh}}}{C_{M_{xh}}}.$$

Following the work of [9,12], who introduced difference-type estimators for median estimation in a two-phase setting, this study extends those formulations to stratified two-phase sampling, defined as:

(14) $${\hat{M}}_{D_{2st}}=\sum_{h=1}^{L}W_{h}\left[d_{2h}{\hat{M}}_{yh}+d_{3h}\left({\overset{´}{M}}_{xh}-{\hat{M}}_{xh}\right)\right],$$

(15) $${\hat{M}}_{D_{3st}}=\sum_{h=1}^{L}W_{h}\left[d_{4h}{\hat{M}}_{yh}+d_{5h}\left({\overset{´}{M}}_{xh}-{\hat{M}}_{xh}\right)\right]\left(\frac{{\overset{´}{M}}_{xh}}{{\hat{M}}_{xh}}\right),$$

(16) $${\hat{M}}_{D_{4st}}=\sum_{h=1}^{L}W_{h}\left[d_{6h}{\hat{M}}_{yh}+d_{7h}\left({\overset{´}{M}}_{xh}-{\hat{M}}_{xh}\right)\right]\left(\frac{{\overset{´}{M}}_{xh}-{\hat{M}}_{xh}}{{\overset{´}{M}}_{xh}+{\hat{M}}_{xh}}\right).$$

The estimators $\left({\hat{D}}_{2st},{\hat{D}}_{3st},{\hat{D}}_{4st}\right)$ achieve their minimum biases and mean squared errors under first-order approximation, at the optimal values, which are given as:

(17) $$Bias{\left({\hat{M}}_{D_{2st}}\right)}_{min}≅-\sum_{h=1}^{L}W_{h}M_{yh}\left[\frac{\theta_{1h}C_{M_{yh}}^{2}C_{M_{xh}}^{2}-\theta_{3h}C_{M_{yxh}}^{2}}{C_{M_{xh}}^{2}\left(1+\theta_{1h}C_{M_{yh}}^{2}\right)-\theta_{3h}C_{M_{yxh}}^{2}}\right],$$

(18) $$Bias{\left({\hat{M}}_{D_{3st}}\right)}_{min}≅\sum_{h=1}^{L}W_{h}M_{yh}\left[\left(d_{4h(opt)}-1\right)+\theta_{3h}\left\{d_{4h(opt)}M_{yh}\left(C_{M_{xh}}^{2}-C_{M_{yxh}}\right)+d_{5h(opt)}M_{xh}C_{M_{xh}}^{2}\right\}\right],$$

(19) $$Bias{\left({\hat{M}}_{D_{4st}}\right)}_{min}≅\sum_{h=1}^{L}W_{h}M_{yh}\left[-1+\frac{\theta_{3h}}{4}C_{M_{xh}}^{2}\left\{-1+d_{6h(opt)}\left(\frac{M_{xh}+8\rho_{yxh}M_{yh}}{M_{xh}}\right)\right\}\right],$$

(20) $$MSE{\left({\hat{M}}_{D_{2st}}\right)}_{min}≅\sum_{h=1}^{L}W_{h}^{2}M_{yh}^{2}\left[\frac{\theta_{1h}C_{M_{yh}}^{2}C_{M_{xh}}^{2}-\theta_{3h}C_{M_{yxh}}^{2}}{C_{M_{xh}}^{2}\left(1+\theta_{1h}C_{M_{yh}}^{2}\right)-\theta_{3h}C_{M_{yxh}}^{2}}\right],$$

(21) $$\begin{array}{cc} MSE{\left({\hat{M}}_{D_{3st}}\right)}_{\min}≅\;\sum_{h=1}^{L}W_{h}^{2}\; & [Bias{\left({\hat{M}}_{D_{3st}}\right)}_{\min}^{2}+\theta_{1h}M_{yh}^{2}d_{4h(\mathrm{opt})}^{2}C_{M_{yh}}^{2}+\theta_{3h}\left\{C_{M_{xh}}^{2}(d_{4h(\mathrm{opt})}M_{yh}\right.\\ \; & \left.+d_{5h(\mathrm{opt})}M_{xh}{)}^{2}-2d_{4h(\mathrm{opt})}M_{yh}C_{M_{yxh}}\left(d_{4h(\mathrm{opt})}M_{yh}+d_{5h(\mathrm{opt})}M_{x}\right)\right\}] \end{array}$$

and

(22) $$MSE{\left({\hat{M}}_{D_{4st}}\right)}_{min}≅\sum_{h=1}^{L}W_{h}^{2}\left[Bias{\left({\hat{M}}_{D_{4st}}\right)}_{min}+\frac{1}{4}\theta_{3h}d_{6h(opt)}^{2}M_{yh}^{2}C_{M_{xh}}^{2}\right],$$

where

$$d_{2h(opt)}=\frac{C_{M_{xh}}^{2}}{C_{M_{xh}}^{2}\left(1+\theta_{1h}C_{M_{yh}}^{2}\right)-\theta_{3h}C_{M_{yxh}}^{2}},$$

$$d_{3h(opt)}=\frac{M_{yh}C_{M_{yxh}}}{M_{xh}\left[C_{M_{xh}}^{2}\left(1+\theta_{1h}C_{M_{yh}}^{2}\right)-\theta_{3h}C_{M_{yxh}}^{2}\right]},$$

$$d_{4h(opt)}=\left[\frac{C_{M_{xh}}^{2}}{C_{M_{xh}}^{2}\left(1+\theta_{1h}C_{M_{yh}}^{2}\left\{1+\theta_{3h}C_{M_{xh}}^{2}\right\}\right)+\theta_{3h}C_{M_{yxh}}^{2}\left(1+C_{M_{xh}}^{2}\right)}\right],$$

$$d_{5h(opt)}=M_{yh}\left[\frac{C_{M_{xh}}^{2}\left(\theta_{1h}C_{M_{yh}}^{2}-1\right)+C_{M_{yxh}}\left(1+-\theta_{3h}C_{M_{yxh}}\right)}{C_{M_{xh}}^{2}\left(1+\theta_{1h}C_{M_{yh}}^{2}\left\{1+\theta_{3h}C_{M_{xh}}^{2}\right\}\right)+\theta_{3h}C_{M_{yxh}}^{2}\left(1+C_{M_{xh}}^{2}\right)}\right],$$

$$d_{6h(opt)}=\frac{1}{8}\left[\frac{8-\theta_{2h}C_{M_{xh}}^{2}}{1+\theta_{1h}C_{M_{xh}}^{2}\left(1-\rho_{yxh}^{2}\right)}\right],$$

and

$$d_{7h\left(opt\right)}=\frac{M_{yh}}{M_{xh}}\left[\frac{1}{2}+d_{6h\left(opt\right)}\left(\frac{\rho_{yxh}M_{yh}}{M_{xh}}\right)-1\right].$$

## 4. A New Stratified Family of Estimators

Inspired by the modified forms of estimators proposed by [28], we introduce a class of stratified transformation-based double exponential estimators within a two-phase stratified random sampling design. The framework strengthens estimation accuracy by combining the stability of a robust central tendency measure with the efficiency derived from auxiliary information obtained during the first phase. This combination provides a practical and reliable tool for modern survey research, where complex populations rarely follow theoretical assumptions. A stratified transformed double exponential family of estimators is expressed as follows:

(23) $${\hat{E}}_{st}=\sum_{h=1}^{L}W_{h}{\hat{M}}_{yh}\exp\left[V_{1h}\left\{\frac{t_{1h}\left({\hat{M}}_{xh}-{\overset{´}{M}}_{xh}\right)}{t_{1h}\left({\overset{´}{M}}_{xh}+{\hat{M}}_{xh}\right)+2t_{2h}}\right\}\right]\exp\left[V_{2h}\left\{\frac{t_{3h}\left({\overset{´}{M}}_{xh}-{\hat{M}}_{xh}\right)}{t_{3h}\left({\overset{´}{M}}_{xh}+{\hat{M}}_{xh}\right)+2t_{4h}}\right\}\right],$$

where $\left(V_{ih},i=1,2\right)$ be predetermined constants. The symbols $t_{1h},t_{2h},t_{3h},t_{4h}$ correspond to known characteristics of the population related to the auxiliary variable *X*. Using Equation (23) as a basis, new estimators are derived through different selections of these quantities. The chosen parameter values are specified in Table 2, and the resulting estimator formulations are displayed in Table 3.

where

$$IQR_{h}=Q_{3h}-Q_{1h},$$

$$MR_{h}=\frac{X_{hmax}+X_{hmin}}{2},$$

$$QA_{h}=\frac{Q_{3h}+Q_{1h}}{2},$$

$$QD_{h}=\frac{Q_{3h}-Q_{1h}}{2},$$

$$TM_{h}=\frac{Q_{1h}+2Q_{2h}+Q_{3h}}{4},$$

$$DM_{h}=\frac{\sum_{i=1}^{9}D_{ih}}{9},$$

$$MAD_{h}=\mathrm{median}\left(\;\left|X_{ih}-M_{xh}\right|\;:\;i=1,\ldots ,N_{h}\right),$$

$$\sigma_{X_{h}}=\sqrt{\frac{1}{N_{h}}\sum_{i=1}^{N_{h}}{\left(X_{ih}-\bar{X_{h}}\right)}^{2}},$$

$$Sk\left(X_{h}\right)=\frac{\frac{1}{N_{h}}\sum_{i=1}^{N_{h}}{\left(X_{ih}-\bar{X_{h}}\right)}^{3}}{\sigma_{X_{h}}^{3}},$$

$$PM=\sqrt{Q_{1h}Q_{3h}}.$$

**Table 3:** Generalized family of estimators under stratified two-phase sampling.

${\hat{E}}_{1st}=\sum_{h=1}^{L}W_{h}{\hat{M}}_{yh}\exp\left[V_{1h}\left\{\frac{QD_{h}\left({\hat{M}}_{xh}-{\overset{´}{M}}_{xh}\right)}{QD_{h}\left({\overset{´}{M}}_{xh}+{\hat{M}}_{xh}\right)+2MAD_{h}}\right\}\right]\exp\left[V_{2h}\left\{\frac{\left({\overset{´}{M}}_{xh}-{\hat{M}}_{xh}\right)}{\left({\overset{´}{M}}_{xh}+{\hat{M}}_{xh}\right)+2\left(X_{hmax}-X_{hmin}\right)}\right\}\right]$
${\hat{E}}_{2st}=\sum_{h=1}^{L}W_{h}{\hat{M}}_{yh}\exp\left[V_{1h}\left\{\frac{TM_{h}\left({\hat{M}}_{xh}-{\overset{´}{M}}_{xh}\right)}{TM_{h}\left({\overset{´}{M}}_{xh}+{\hat{M}}_{xh}\right)+2MR_{h}}\right\}\right]\exp\left[V_{2h}\left\{\frac{\left({\overset{´}{M}}_{xh}-{\hat{M}}_{xh}\right)}{\left({\overset{´}{M}}_{xh}+{\hat{M}}_{xh}\right)+2IQR_{h}}\right\}\right]$
${\hat{E}}_{3st}=\sum_{h=1}^{L}W_{h}{\hat{M}}_{yh}\exp\left[V_{1h}\left\{\frac{DM_{h}\left({\hat{M}}_{xh}-{\overset{´}{M}}_{xh}\right)}{DM_{h}\left({\overset{´}{M}}_{xh}+{\hat{M}}_{xh}\right)+2MAD_{h}}\right\}\right]\exp\left[V_{2h}\left\{\frac{\left({\overset{´}{M}}_{xh}-{\hat{M}}_{xh}\right)}{\left({\overset{´}{M}}_{xh}+{\hat{M}}_{xh}\right)+2QD_{h}}\right\}\right]$
${\hat{E}}_{4st}=\sum_{h=1}^{L}W_{h}{\hat{M}}_{yh}\exp\left[V_{1h}\left\{\frac{Sk\left(X_{h}\right)\left({\hat{M}}_{xh}-{\overset{´}{M}}_{xh}\right)}{Sk\left(X_{h}\right)\left({\overset{´}{M}}_{xh}+{\hat{M}}_{xh}\right)+2}\right\}\right]\exp\left[V_{2h}\left\{\frac{\left({\overset{´}{M}}_{xh}-{\hat{M}}_{xh}\right)}{\left({\overset{´}{M}}_{xh}+{\hat{M}}_{xh}\right)+2\left(Q_{3h}-Q_{2h}\right)}\right\}\right]$
${\hat{E}}_{5st}=\sum_{h=1}^{L}W_{h}{\hat{M}}_{yh}\exp\left[V_{1h}\left\{\frac{\log\left(Q_{3h}-1\right)\left({\hat{M}}_{xh}-{\overset{´}{M}}_{xh}\right)}{log\left(Q_{3h}-1\right)\left({\overset{´}{M}}_{xh}+{\hat{M}}_{xh}\right)+2\log\left(Q_{1h}-1\right)}\right\}\right]\exp\left[V_{2h}\left\{\frac{\left({\overset{´}{M}}_{xh}-{\hat{M}}_{xh}\right)}{\left({\overset{´}{M}}_{xh}+{\hat{M}}_{xh}\right)+2\log\left(MR_{h}-1\right)}\right\}\right]$
${\hat{E}}_{6st}=\sum_{h=1}^{L}W_{h}{\hat{M}}_{yh}\exp\left[V_{1h}\left\{\frac{QA_{h}\left({\hat{M}}_{xh}-{\overset{´}{M}}_{xh}\right)}{QA_{h}\left({\overset{´}{M}}_{xh}+{\hat{M}}_{xh}\right)+2\sigma_{X_{h}}}\right\}\right]\exp\left[V_{2h}\left\{\frac{\left({\overset{´}{M}}_{xh}-{\hat{M}}_{xh}\right)}{\left({\overset{´}{M}}_{xh}+{\hat{M}}_{xh}\right)+2QD_{h}}\right\}\right]$
${\hat{E}}_{7st}=\sum_{h=1}^{L}W_{h}{\hat{M}}_{yh}\exp\left[V_{1h}\left\{\frac{X_{hmedian}\left({\hat{M}}_{xh}-{\overset{´}{M}}_{xh}\right)}{X_{hmedian}\left({\overset{´}{M}}_{xh}+{\hat{M}}_{xh}\right)+2QR_{h}}\right\}\right]\exp\left[V_{2h}\left\{\frac{\left({\overset{´}{M}}_{xh}-{\hat{M}}_{xh}\right)}{\left({\overset{´}{M}}_{xh}+{\hat{M}}_{xh}\right)+2IQR_{h}}\right\}\right]$
${\hat{E}}_{8st}=\sum_{h=1}^{L}W_{h}{\hat{M}}_{yh}\exp\left[V_{1h}\left\{\frac{\sqrt{Q_{1h}Q_{3h}}\left({\hat{M}}_{xh}-{\overset{´}{M}}_{xh}\right)}{\sqrt{Q_{1h}Q_{3h}}\left({\overset{´}{M}}_{xh}+{\hat{M}}_{xh}\right)+2\sqrt{X_{hmax}X_{hmin}}}\right\}\right]\exp\left[V_{2h}\left\{\frac{\left({\overset{´}{M}}_{xh}-{\hat{M}}_{xh}\right)}{\left({\overset{´}{M}}_{xh}+{\hat{M}}_{xh}\right)+2\sqrt{IQR_{h}}}\right\}\right]$

### 4.1. Conceptual Explanation of the Proposed Transformations

The calibration constants $t_{ih}$ and $V_{ih}$ control how auxiliary data influences the estimation exponential structure. Moment-based measures, which are commonly used in classical exponential estimators, can prove ineffective when the population is skew or contains extreme values. By introducing quantile-based scaling quantities, the suggested framework, on the other hand, provides continuity and resistance against heterogeneous strata, asymmetric distributions, and heavy tails. A significant distributional feature, such as median-centered spread, tail behavior, or resistance to extreme observations, is reflected in each chosen transformation. Because of this, the auxiliary variable improves efficiency and maintains robustness for median estimation under stratified two-phase sampling by providing distributionally significant data even in the presence of deviations from normality.

### 4.2. Underlying Logic for Each Quantile Measure Used in the Methodology

- **Quartile deviation (QD):** When variation increases by outliers, the quartile deviation (QD) is a useful tool for capturing variability around the median.
- **Median absolute deviation (MAD):** A highly effective scale measure that reduces the impact of extreme observations is the median absolute deviation (MAD).
- **Trimmed mean (TM):** Reductions in tail values under high skewness to provide a stable location measure.
- **Mid range (MR):** Useful for auxiliary variables with wide range, it reflects the behavior of extreme values.
- **Interquartile range IQR:** The IQR provides moderate efficiency and robustness by summarizing the middle half of the distribution.
- **Decile mean (DM):** The shape of the distribution is summarized and aspects of inequality that go beyond conventional dispersion measures are captured by the decile mean (DM), which is the average of the distribution’s decile values.
- **Skewness measure $\mathrm{Sk}(X_{h})$:** Corrects the estimator for directional asymmetry in each stratum.
- **Quartile average (QA):** A balanced alternative for the median is the quartile average (QA), a smooth measure of central tendency.
- **Product measure (PM):** Product measure represents a balanced distribution around the median, which is particularly helpful in skewed environments.

### 4.3. Remark on the Structure of the Proposed Family

In particular, the suggested class of estimators has eight distinct variants, each represented by ${\hat{E}}_{1st}$ through ${\hat{E}}_{8st}$. Since each estimator is developed using a different quantile-based transformation of the auxiliary variable, they all show different tail behavior and sensitivity to distributional asymmetry. For example, ${\hat{E}}_{1st}$ and ${\hat{E}}_{3st}$ are more resilient to extreme values because they rely on quartile deviation and median absolute deviation, respectively. By using skewness and logarithmic quantile measures, estimators like ${\hat{E}}_{4st}$ and ${\hat{E}}_{5st}$ can more directly adapt to linear asymmetry. A combination of composite quantile summaries like the quartile average and geometric quartile product, ${\hat{E}}_{6st}$–${\hat{E}}_{8st}$ offers a balanced response to both central and tail information. Even when the population deviates greatly from symmetry, this adaptable structure ensures that the family can accommodate different distributional patterns across strata, enabling effective median estimation.

The following theorem presents the mathematical expressions of the bias and mean squared error associated with the ratio–product-type family of estimators ${\hat{E}}_{st}$.

**Theorem 1.** 
*Let ${\hat{E}}_{st}$ denote an exponential ratio–product family of estimators constructed under a stratified two-phase sampling scheme for the estimation of the population median $M_{y}$. The resulting bias and mean squared error (MSE) formulations are presented below:*

$$\begin{array}{c} Bias\left({\hat{E}}_{st}\right)≅\sum_{h=1}^{L}W_{h}M_{yh}\left[\frac{1}{2}\left(k_{1h}-k_{2h}\right)\theta_{3h}C_{M_{yxh}}-\frac{1}{8}\left(k_{1h}^{2}-k_{2h}^{2}-2k_{1h}k_{2h}\right)\theta_{3h}C_{M_{xh}}^{2}\right] \end{array}$$

*and*

$$\begin{array}{c} MSE\left({\hat{E}}_{st}\right)≅\sum_{h=1}^{L}W_{h}^{2}M_{yh}^{2}\left[\theta_{1h}C_{M_{yh}}^{2}+\frac{1}{4}\theta_{3h}C_{M_{xh}}^{2}\left(k_{1h}^{2}k_{2h}^{2}-2k_{1h}k_{2h}\right)+\theta_{3h}C_{M_{yxh}}\left(k_{1h}-k_{2h}\right)\right]. \end{array}$$

**Proof.**  For completeness, the concepts necessary for proving the theorem are summarized below:

$$e_{0h}=\left(\frac{{\hat{M}}_{yh}-M_{yh}}{M_{yh}}\right),$$

$$e_{1h}=\left(\frac{{\hat{M}}_{xh}-M_{xh}}{M_{xh}}\right)$$

and

$$e_{2h}=\left(\frac{{\overset{´}{M}}_{xh}-M_{xh}}{M_{xh}}\right),$$

such that $E\left(e_{ih}\right)=0$ for i=0,1,2.

Moreover,

$$E\left(e_{0h}^{2}\right)=\theta_{1h}C_{M_{yh}}^{2},$$

$$E\left(e_{1h}^{2}\right)=\theta_{1h}C_{M_{xh}}^{2},$$

$$E\left(e_{2h}^{2}\right)=\theta_{2h}C_{M_{xh}}^{2},$$

$$E\left(e_{0h}e_{1h}\right)=\theta_{1h}C_{M_{yxh}}=\rho_{yxh}C_{M_{yh}}C_{M_{xh}},$$

$$E\left(e_{0h}e_{2h}\right)=\theta_{2h}C_{M_{yxh}}=\rho_{yxh}C_{M_{yh}}C_{M_{xh}},$$

$$E\left(e_{1h}e_{2h}\right)=\theta_{2h}C_{M_{xh}}^{2},$$

where

$$C_{M_{yh}}=\frac{1}{M_{yh}f_{yh}\left(M_{yh}\right)},$$

$$C_{M_{xh}}=\frac{1}{M_{yh}f_{xh}\left(M_{xh}\right)},$$

$$\mathrm{Cov}\left(M_{y_{h}},M_{x_{h}}\right)=\frac{\rho_{yxh}}{4f_{yh}\left(M_{yh}\right)f_{xh}\left(M_{xh}\right)}$$

$$\theta_{1h}=\frac{1}{4}\left(\frac{1}{n_{h}}-\frac{1}{N_{h}}\right),\;\theta_{2h}=\frac{1}{4}\left(\frac{1}{m_{h}}-\frac{1}{N_{h}}\right),$$

$$\theta_{3h}=\frac{1}{4}\left(\frac{1}{n_{h}}-\frac{1}{m_{h}}\right).$$

Expressing (23) through relative errors allows us to obtain an analytical expression:

(24) $$\begin{array}{c} {\hat{E}}_{st}=\sum_{h=1}^{L}W_{h}M_{yh}\left(1+e_{0h}\right)\exp\left[V_{1h}\left\{\frac{k_{1h}\left(e_{1h}-e_{2h}\right)}{2}{\left(1+\frac{k_{1h}\left(e_{1h}+e_{2h}\right)}{2}\right)}^{-1}\right\}\right]\times \\ \;\exp\left[V_{2h}\left\{\frac{-k_{2h}\left(e_{1h}-e_{2h}\right)}{2}{\left(1+\frac{k_{2h}\left(e_{1h}+e_{2h}\right)}{2}\right)}^{-1}\right\}\right] \end{array}$$

where $V_{1h},V_{2h},k_{1h},$ and $k_{2h}$ are defined as:

$$V_{1h}=V_{2h}=1,$$

$$k_{1h}=\frac{t_{1h}M_{xh}}{t_{1h}M_{xh}+t_{2h}}$$

and

$$k_{2h}=\frac{t_{3h}M_{xh}}{t_{3h}M_{xh}+t_{4h}}.$$

The right-hand side of Equation (24) is approximated via a first-order Taylor expansion, disregarding higher-order terms $\left(e_{ih}>2\right)$ as their effects are negligible. This yields:

$$\begin{array}{c} {\hat{E}}_{st}=\sum_{h=1}^{L}W_{h}M_{yh}\left(1+e_{0h}\right)\exp\left[\frac{k_{1h}\left(e_{1h}-e_{2h}\right)}{2}\left\{1-\frac{k_{1h}\left(e_{1h}+e_{2h}\right)}{2}+\frac{k_{1h}^{2}{\left(e_{1h}+e_{2h}\right)}^{2}}{4}\right\}\right]\times \\ \;\exp\left[-\frac{k_{2h}\left(e_{1h}-e_{2h}\right)}{2}\left\{1-\frac{k_{2h}\left(e_{1h}+e_{2h}\right)}{2}+\frac{k_{2h}^{2}{\left(e_{1h}+e_{2h}\right)}^{2}}{4}\right\}\right], \end{array}$$

$$\begin{array}{c} {\hat{E}}_{st}=\sum_{h=1}^{L}W_{h}M_{yh}\left(1+e_{0h}\right)\exp\left[\frac{k_{1h}\left(e_{1h}-e_{2h}\right)}{2}-\frac{k_{1h}^{2}\left(e_{1h}^{2}-e_{2h}^{2}\right)}{4}\right]\exp\left[\frac{k_{2h}\left(e_{2h}-e_{1h}\right)}{2}-\frac{k_{2h}^{2}\left(e_{1h}^{2}-e_{2h}^{2}\right)}{4}\right]. \end{array}$$

After simplifying, we obtain:

(25) $$\begin{array}{cc} {\hat{E}}_{st}-\sum_{h=1}^{L}W_{h}M_{yh}\;≅\; & \sum_{h=1}^{L}W_{h}M_{yh}[e_{0h}+\frac{k_{1h}}{2}\left(e_{1h}-e_{2h}+e_{0h}e_{1h}-e_{0h}e_{2h}\right)\\ \; & +\frac{k_{2h}}{2}\left(e_{2h}-e_{1h}-e_{0h}e_{1h}+e_{0h}e_{2h}\right)-\frac{k_{1h}^{2}}{8}\left(e_{1h}^{2}-3e_{2h}^{2}+2e_{1h}e_{2h}\right)\\ \; & -\frac{k_{2h}^{2}}{8}\left(e_{1h}^{2}-3e_{2h}^{2}+2e_{1h}e_{2h}\right)-\frac{k_{1h}k_{2h}}{4}\left(e_{1h}^{2}e_{2h}^{2}-2e_{1h}e_{2h}\right)]. \end{array}$$

The bias of ${\hat{E}}_{st}$ is computed by applying the expectation operator to Equation (25) and replacing each term $e_{0h},e_{1h},e_{1h},e_{0h}^{2},e_{1h}^{2},e_{2h}^{2},e_{0h}e_{1h},e_{0h}e_{2h},e_{1h}e_{2h}$) with its expected value, leading to:

$$\begin{array}{cc} Bias\left({\hat{E}}_{st}\right)≅\sum_{h=1}^{L}W_{h}M_{yh}[ & \frac{1}{2}\left(k_{1h}-k_{2h}\right)\left(\theta_{1h}C_{M_{yxh}}-\theta_{2h}C_{M_{yxh}}\right)-\frac{1}{8}\left(k_{1h}^{2}-k_{2h}^{2}\right)\times \\ & \left(\theta_{1h}C_{M_{xh}}^{2}-\theta_{2h}C_{M_{xh}}^{2}\right)-\frac{k_{1h}k_{2h}}{4}\left(\theta_{1h}C_{M_{xh}}^{2}-\theta_{2h}C_{M_{xh}}^{2}\right)]. \end{array}$$

After simplification, we get:

(26) $$\begin{array}{c} Bias\left({\hat{E}}_{st}\right)≅\sum_{h=1}^{L}W_{h}M_{yh}\left[\frac{1}{2}\left(k_{1h}-k_{2h}\right)\theta_{3h}C_{M_{yxh}}-\frac{1}{8}\left(k_{1h}^{2}-k_{2h}^{2}-2k_{1h}k_{2h}\right)\theta_{3h}C_{M_{xh}}^{2}\right], \end{array}$$

where

$$\theta_{3h}=\theta_{1h}-\theta_{2h}.$$

Under a first-order approximation, the MSE of ${\hat{E}}_{st}$ is derived by squaring Equation (25) and taking expectations, keeping only terms up to second order in ${e_{h}}^{\prime }s$:

(27) $$\begin{array}{c} MSE\left({\hat{E}}_{st}\right)≅\sum_{h=1}^{L}W_{h}^{2}M_{yh}^{2}\left[\theta_{1h}C_{M_{yh}}^{2}+\frac{1}{4}\theta_{3h}C_{M_{xh}}^{2}\left(k_{1h}^{2}+k_{2h}^{2}-2k_{1h}k_{2h}\right)+\theta_{3h}C_{M_{yxh}}\left(k_{1h}-k_{2h}\right)\right]. \end{array}$$

□

## 5. Evaluation Framework and Conditions

From the MSE formulation of the proposed estimator (Theorem 1, Equation (27)),

$$\begin{array}{c} MSE\left({\hat{E}}_{st}\right)≅\sum_{h=1}^{L}W_{h}^{2}M_{yh}^{2}\left[\theta_{1h}C_{M_{yh}}^{2}+\frac{1}{4}\theta_{3h}C_{M_{xh}}^{2}\left(k_{1h}^{2}+k_{2h}^{2}-2k_{1h}k_{2h}\right)+\theta_{3h}C_{M_{yxh}}\left(k_{1h}-k_{2h}\right)\right]. \end{array}$$

the superiority of ${\hat{E}}_{st}$ over its counterparts in Section 2 is ensured under the following inequalities.

(**i**) Upon comparing the MSE expression derived for the proposed estimator family (27) with the corresponding variance of the sample median (2), the condition stated below is obtained:
$$Var\left({\hat{M}}_{yst}\right)>MSE\left({\hat{E}}_{st}\right)\mathrm{if}$$
$$\sum_{h=1}^{L}\theta_{3h}W_{h}^{2}M_{yh}^{2}\left[C_{M_{xh}}^{2}\left(k_{1h}^{2}+k_{2h}^{2}-2k_{1h}k_{2h}\right)+4C_{M_{yxh}}\left(k_{1h}-k_{2h}\right)\right]>0.$$
(**ii**) When the MSE from Equation (27) is compared with that from Equation (5), the following condition is derived:
$$MSE\left({\hat{M}}_{Ast}\right)>MSE\left({\hat{E}}_{st}\right)\mathrm{if}$$
$$4\sum_{h=1}^{L}W_{h}^{2}M_{yh}^{2}C_{M_{xh}}^{2}>\sum_{h=1}^{L}\theta_{3h}W_{h}^{2}M_{yh}^{2}\left[C_{M_{xh}}^{2}\left(k_{1h}^{2}k_{2h}^{2}-2k_{1h}k_{2h}\right)+\theta_{3h}C_{M_{yxh}}\left(k_{1h}-k_{2h}+2\right)\right].$$
(**iii**) A specific condition can be derived by evaluating the MSE of the estimators from Equation (27) against the MSE presented in Equation (7):
$$MSE{\left({\hat{M}}_{D_{1st}}\right)}_{min}>MSE\left({\hat{E}}_{st}\right)\mathrm{if}$$
$$\sum_{h=1}^{L}\theta_{3h}W_{h}^{2}M_{yh}^{2}\left[C_{M_{xh}}^{2}\left(k_{1h}^{2}+k_{2h}^{2}-2k_{1h}k_{2h}\right)+4C_{M_{yxh}}\left(k_{1h}-k_{2h}\right)\right]>4\sum_{h=1}^{L}\theta_{3h}W_{h}^{2}M_{yh}^{2}C_{M_{yh}}^{2}\rho_{yxh}^{2}.$$
(**iv**) A specific condition follows from evaluating the MSE of the estimators in Equation (27) against that in Equation (12):
$$MSE\left({\hat{M}}_{Rest}\right)>MSE\left({\hat{E}}_{st}\right)\mathrm{if}$$
$$\sum_{h=1}^{L}\theta_{3h}W_{h}^{2}M_{yh}^{2}C_{M_{xh}}^{2}\left(1-4K_{h}\right)>\sum_{h=1}^{L}\theta_{3h}W_{h}^{2}M_{yh}^{2}\left[C_{M_{xh}}^{2}\left(k_{1h}^{2}+k_{2h}^{2}-2k_{1h}k_{2h}\right)+4C_{M_{yxh}}\left(k_{1h}-k_{2h}\right)\right].$$
(**v**) The following condition is derived by examining the mean squared error of the estimators in Equation (27) alongside the MSE provided in Equation (13):
$$MSE\left({\hat{M}}_{Pest}\right)>MSE\left({\hat{E}}_{st}\right)\mathrm{if}$$
$$\sum_{h=1}^{L}\theta_{3h}W_{h}^{2}M_{yh}^{2}C_{M_{xh}}^{2}\left(1+4K_{h}\right)>\sum_{h=1}^{L}\theta_{3h}W_{h}^{2}M_{yh}^{2}\left[C_{M_{xh}}^{2}\left(k_{1h}^{2}+k_{2h}^{2}-2k_{1h}k_{2h}\right)+4C_{M_{yxh}}\left(k_{1h}-k_{2h}\right)\right].$$
(**vi**) The MSE of the proposed estimator family, as expressed in Equation (27), is examined to establish the following condition:
  $$MSE{\left({\hat{M}}_{D_{2st}}\right)}_{\min}>MSE\left({\hat{E}}_{st}\right)\mathrm{if}$$
  $$\sum_{h=1}^{L}W_{h}^{2}M_{yh}^{2}\left[\frac{\left(1-R_{h}\right)\left(\theta_{1h}C_{M_{yh}}^{2}C_{M_{xh}}^{2}-\theta_{3h}C_{M_{yxh}}^{2}\right)-R_{h}C_{M_{xh}}^{2}}{C_{M_{xh}}^{2}\left(1+\theta_{1h}C_{M_{yh}}^{2}\right)-\theta_{3h}C_{M_{yxh}}^{2}}\right]>0,$$
  where
  $$\theta_{1h}C_{M_{yh}}^{2}+\frac{1}{4}\theta_{3h}C_{M_{xh}}^{2}\left(k_{1h}^{2}k_{2h}^{2}-2k_{1h}k_{2h}\right)+\theta_{3h}C_{M_{yxh}}\left(k_{1h}-k_{2h}\right).$$
(**vii**) A comparison between the MSE of the new estimator family in (27) and MSE ${\hat{M}}_{D_{3st}}$ yields the following condition:
  $$MSE{\left({\hat{M}}_{D_{3st}}\right)}_{\min}>MSE\left({\hat{E}}_{st}\right)\mathrm{if}$$
  $$4\sum_{h=1}^{L}W_{h}^{2}\left(B_{D_{3h}}+\Psi_{D_{3h}}\right)>\sum_{h=1}^{L}\theta_{3h}W_{h}^{2}M_{yh}^{2}\left[C_{M_{xh}}^{2}\left(k_{1h}^{2}k_{2h}^{2}-2k_{1h}k_{2h}\right)+4C_{M_{yxh}}\left(k_{1h}-k_{2h}\right)\right],$$
  where
  $$\Psi_{D_{3h}}=\theta_{3h}\left\{C_{M_{xh}}^{2}{\left(d_{4h(opt)}M_{yh}+d_{5h(opt)}M_{xh}\right)}^{2}-2\;d_{4h(opt)}M_{yh}\;C_{M_{yxh}}\left(d_{4h(opt)}M_{yh}+d_{5h(opt)}M_{xh}\right)\right\}$$
  and
  $$B_{D_{3h}}=Bias{\left({\hat{M}}_{D_{3st}}\right)}_{min}^{2}+\theta_{1h}M_{yh}^{2}\left(d_{4h(opt)}^{2}-1\right)C_{M_{yh}}^{2}.$$
(**viii**) A specific condition is derived by analyzing the mean squared error of the estimators expressed in Equation (27) against the MSE formulation in Equation (22):
  $$MSE{\left({\hat{M}}_{D_{4st}}\right)}_{\min}>MSE\left({\hat{E}}_{st}\right)\mathrm{if}$$
  $$4\sum_{h=1}^{L}W_{h}^{2}M_{yh}^{2}\left[B_{D_{4st}}+\frac{\theta_{3h}}{4}d_{6h(opt)}^{2}C_{M_{xh}}^{2}-\theta_{1h}C_{M_{yh}}^{2}\right]>\sum_{h=1}^{L}\theta_{3h}W_{h}^{2}M_{yh}^{2}Z_{h},$$
  where
  $$B_{D_{4st}}=\frac{Bias{\left({\hat{M}}_{D_{4st}}\right)}_{min}}{M_{yh}^{2}}$$
  and
  $$Z_{h}=C_{M_{xh}}^{2}\left(k_{1h}^{2}k_{2h}^{2}-2k_{1h}k_{2h}\right)+4C_{M_{yxh}}\left(k_{1h}-k_{2h}\right).$$

### Practical Feasibility and Computational Considerations

The quantile-based measures suggested for use in this study, including the median absolute deviation, interquartile range, and trimmed mean, are practically computable even in substantial first-phase samples. Modern statistical applications and fast algorithms make it easy to quickly calculate these measures with little processing time. Even though it takes a little more work to calculate them than traditional mean or variance-based methods, the extra cost is small compared to the cost of collecting all the data. More importantly, these strong measures give stable and consistent results even when there are outliers or skewed distributions, which is where simpler measures often fail. Hence, the modest increase in computational effort is justified by the significant improvement in robustness, precision, and reliability that these methods offer in stratified two-phase.

## 6. Analysis of Results

The performance of the proposed estimators is investigated through a combination of simulation and empirical analysis. Efficiency in various tightly controlled scenarios can be obtained from simulations of populations set up under positively skewed distributional forms. Further evidence from actual datasets is added to these findings to ensure that the conclusions are both theoretically sound and practically applicable.

### 6.1. Simulation Study

A comprehensive simulation study was conducted under stratified two-phase sampling to assess the finite population parameters and behavior of the proposed estimators. The simulations studied populations derived from various non-normal distributions, such as heavy-tailed, asymmetric, and moderately skewed models. Different combinations of first-phase and second-phase sample sizes were examined to reflect practical survey conditions. For each design, the empirical mean squared error and percent relative efficiency values are calculated through repeated sampling to analyze estimation accuracy. To demonstrate the effectiveness and strength of the suggested estimators, we compared their performance to the outcomes of a number of existing techniques. This setup gives us a controlled scenario to show how the new estimators work with different types of distributional structures and sampling schemes.

In statistical analysis, the choice of distributions to use in simulation studies should represent conditions in which the estimators will be used. As a robust estimator, the median works well when the data have skewness, heavy tails, or other irregular distributions. The study uses the auxiliary variable X to test its behavior in such a situation by constructing five representative distributions, with each distribution being selected because of its distributional properties.

- **Population 1:** The first population assumes a heavy-tailed Cauchy distribution for *X*, specified with location $\lambda_{1}=21$ and scale $\lambda_{2}=16$. The association between *X* and *Y* is negative, fixed at −0.40.
- **Population 2:** In the second case, *X* is uniformly distributed between 17 and 24. This distribution is considered independent of *Y*, i.e., no correlation is introduced.
- **Population 3:** For the third population, *X* is modeled by an exponential distribution with parameter $\lambda_{5}=0.5$, capturing a strong right-skew. The correlation with *Y* is set at 0.60.
- **Population 4:** The fourth design specifies *X* as following a gamma distribution, parameterized by $\lambda_{6}=23$ and $\lambda_{7}=15$. The correlation with *Y* is moderately strong, at 0.68.
- **Population 5:** Finally, the fifth population generates *X* from a log-normal distribution with parameters 11 and 6, representing a mildly skewed distribution. A correlation of 0.57 is introduced with *Y*.

In all populations, the outcome variable is expressed as

$$Y=\rho_{yx}X+e,$$

where *e* is drawn from a standard normal distribution, ensuring that randomness is properly incorporated.

The study compared the MSE of the proposed estimators with those of existing alternatives across different distributions and correlation levels. The procedures adopted are consistent with the approaches presented in [28]. All calculations were carried out using R software (latest v. 4.4.0), ensuring a systematic assessment of robustness and efficiency within the stratified two-phase sampling scheme.

**Step 1:** As part of the simulation design, a finite population consisting of N=1000 units is generated for the variables *X* and Y. The population is partitioned into *L* strata, which are defined either through prior knowledge of strata boundaries or through the use of an auxiliary variable.
**Step 2:** The first stage of sampling involves drawing a stratified sample of total size *m*. For each stratum *h*th (h=1,2,…,L), a subsample of size $m_{h}$ is selected using SRSWOR. The distribution of $m_{h}$ across strata is arranged by fixed-quota allocation rules.
**Step 3:** Following the first-phase stratified sampling, a second-phase subsample comprising *n* total observations is selected. Within each stratum *h*, $n_{h}$ units are drawn from the $m_{h}$ first-phase units using SRSWOR.
**Step 4:** Consistent with the two-phase design, multiple (m,n) settings are examined with n<m<N. For each pair, the stratum-specific allocations $\left\{m_{h},n_{h}\right\}$ are assigned based on the selected allocation strategy.

- **Scheme 1**: The total first-phase sample size *m* takes values 300, 500, 800, and for each *m*, the second-phase size *n* assumes 0.10 m, 0.20 m, 0.30 m, 0.40 m (rounded). Both $m_{h}$ and $n_{h}$ are equally distributed across all strata.
- **Scheme 2**: Four (m,n) pairs (200,50), (300,75), (500,125), and (800,200) are examined, keeping equal stratum allocation.
- **Scheme 3**: A finer set of designs combines m∈ 150, 250, 400, 600, 900 with n∈ 0.10 m, 0.25 m, 0.40 m, deriving $m_{h}$ and $n_{h}$ by equal allocation across strata.

**Step 5:** The efficiency of the estimators is examined by deriving the necessary stratum-level statistics from the selected samples in accordance with the previously outlined methodology. In the case of existing estimators that depend on unknown constants, the corresponding parameters are optimized using stratified estimates.
**Step 6 (Simulation of stratified samples):** For each population and each chosen (m,n):
  1. Per-stratum allocations $\left\{m_{h},n_{h}\right\}$ are determined according to the allocation rule.
  2. From each stratum *h*, $m_{h}$ units are drawn from the $N_{h}$ population units by SRSWOR (first phase).
  3. From the $m_{h}$ first-phase units in each stratum, $n_{h}$ units are drawn by SRSWOR (second phase).
**Step 7:** For each pairing of (m,n) and for all estimators, the MSE is evaluated using the stratified sampling design. This involves applying the design weights together with the stratum-level statistics obtained from the sampled data.
**Step 8:** To obtain reliable results, the sampling process is repeated 20,000 times. For every estimator and (m,n) arrangement, the mean squared error (MSE) is then computed as the average over these replications. The empirical MSE values for each estimator are obtained as:
  $$MSE{\left({\hat{M}}_{t}\right)}_{\min}\;=\frac{\sum_{u=1}^{U}{\left({\hat{M}}_{tu}-M_{y}\right)}^{2}}{U}$$
  and
  $$\mathrm{PRE}=100\times \frac{\mathrm{Var}({\hat{M}}_{yst})}{\mathrm{MSE}{\left({\hat{M}}_{t}\right)}_{\min}}$$
  where *t* ($t=y_{st},\;A_{st},\;D_{1st}\;,\;Re_{st},\;Pe_{st},\;D_{2st},\;D_{3st}\;D_{4st},\;E_{st1},\;E_{st2},\ldots ,E_{st8}$), U=20,000, ${\hat{M}}_{ut}$ is the estimate from replication *u*, and $M_{y}$ is the true population parameter.

The proposed estimators showed the greatest improvements when the underlying population exhibited skewness or heavy tails. This behaviour is expected because the methodology utilizes quantile based information that remains stable under extreme values and asymmetric shapes. Traditional estimators that depend on means or variance measures are more sensitive to outlying observations, which can distort median estimation in irregular populations. By contrast, the quantile driven structure used here captures central tendency and scale in a way that reflects the true distributional form, leading to superior precision in these challenging settings.

### 6.2. Real-Life Application

This section reports an empirical study utilizing real population data, with the key characteristics of the datasets summarized below. These datasets serve as practical benchmarks, allowing the proposed estimators to be evaluated under varied and representative conditions.

**Population 1.** This information collected from [32], which provides comprehensive details on government schools for the academic year 2012–2013, is used for empirical evaluation. Primary and middle school enrollment data by gender represents the population. In particular, $X_{1}$ denotes the total number of government primary schools for both boys and girls, while $Y_{1}$ represents the total number of enrolled students. Simultaneously, $X_{2}$ represents the total number of government-run middle schools that accept both genders, while $Y_{2}$ records the overall number of students enrolled. It is accessible for download using the following URL: https://repository.lahoreschool.edu.pk/xmlui/bitstream/handle/123456789/13900/Dev-2014.pdf?sequence=1&isAllowed=y (accessed on 28 September 2025). The summary statistics is obtained as:
  $$\begin{array}{c} N_{1}=36,m_{1}=18,n_{1}=9,N_{2}=36,m_{2}=18,n_{2}=9,X_{1_{min}}=388,X_{1_{max}}=1534,X_{2_{min}}=84,\\ X_{2_{max}}=478,M_{x_{1}}=1016.500,M_{y_{1}}=116230,\sigma_{x_{1}}=402.609,\sigma_{x_{2}}=424.937,M_{x_{2}}=206,M_{y_{2}}=\\ 49661,f_{x_{1}}\left(M_{x_{1}}\right)=0.000951993,f_{y_{1}}\left(M_{y_{1}}\right)=0.00000835,f_{x_{2}}\left(M_{x_{2}}\right)=0.004094403,f_{y_{2}}\left(M_{y_{2}}\right)=\\ 0.0000143374,\rho_{y_{1}x_{1}}=0.084,\rho_{y_{2}x_{2}}=0.875,TM_{1}=891.188,TM_{2}=210.688,DM_{1}=982.650\\ ,DM_{2}=231,Sk_{1}=1.008,Sk_{2}=1.023,QA_{1}=891.875,QA_{2}=215.375,QD_{1}=982.650,QD_{2}=\\ 62.875,MAD_{1}=289,MAD_{2}=267,QR_{1}=378.250,QR_{2}=125.750,MR_{1}=961,MR_{R_{2}}=281. \end{array}$$
**Population 2.** A finite population is examined using statistics from [33] to demonstrate the empirical performance of the suggested estimators. Information on industrial activity, particularly the number of registered factories and related employment levels, is provided by these data at the district and division levels. In this case, $X_{1}$ represents the total number of factories registered in 2010, while $Y_{1}$ represents employment by division and district in 2010. The stated employment levels for 2012 are represented by $Y_{2}$, while the number of registered factories is represented by $X_{2}$. The following website provides a download link: https://repository.lahoreschool.edu.pk/xmlui/bitstream/handle/123456789/13023/2013.pdf?sequence=1&isAllowed=y (accessed on 28 September 2025). The summary statistics is presented as:
  $$\begin{array}{c} N_{1}=36,m_{1}=18,n_{1}=9,N_{2}=36,m_{2}=18,n_{2}=9,X_{1_{min}}=24,X_{1_{max}}=1986,X_{2_{min}}=24,\\ X_{2_{max}}=2055,M_{x_{1}}=168.500,M_{y_{1}}=10484.500,\sigma_{1}=438.519,\sigma_{2}=452.713,M_{x_{2}}=171.500,\\ M_{y_{2}}=10494.500,f_{x_{1}}\left(M_{x_{1}}\right)=0.002463666,f_{y_{1}}\left(M_{y_{1}}\right)=0.00004033736,f_{x_{2}}\left(M_{x_{2}}\right)=0.002315051,\\ f_{y_{2}}\left(M_{y_{2}}\right)=0.00004086913,\rho_{y_{1}x_{1}}=0.912,\rho_{y_{2}x_{2}}=0.5194465,T_{M_{1}}=193.438,T_{M_{2}}=195.750,\\ DM_{1}=432.500,DM_{2}=431.500,Sk_{1}=2.106,Sk_{2}=2.345,QA_{1}=218.375,QA_{2}=220,\\ QD_{1}=127.125,QD_{2}=132.500,MAD_{1}=193.438,MAD_{2}=99,QR_{1}=252.25,QR_{2}=265,\\ MR_{1}=1005,MR_{2}=1039.500. \end{array}$$
**Population 3.** The data on page [1] illustrates the amount of money people earn and spend on food. Here, *Y* represents the family’s food expenses, which vary according to their employment status and demonstrate how work can impact food expenditures. Weekly income is reflected in the variable *X*, providing a quick overview of the household’s financial situation. The data is divided into two groups, and the statistics are summarized as follows:
  $$\begin{array}{c} N_{1}=36,m_{1}=18,n_{1}=9,N_{2}=36,m_{2}=18,n_{2}=9,X_{1_{min}}=28,X_{1_{max}}=95,X_{2_{min}}=15,\\ X_{2_{max}}=75,M_{x_{1}}=60.664,M_{y_{1}}=52.728,\sigma_{1}=10.130,\sigma_{2}=12.750,M_{x_{2}}=44.205,M_{y_{2}}=\\ 46.339,f_{x_{1}}\left(M_{x_{1}}\right)=0.0004729,f_{y_{1}}\left(M_{y_{1}}\right)=0.0000219,f_{x_{2}}\left(M_{x_{2}}\right)=0.0003791,f_{y_{2}}\left(M_{y_{2}}\right)=\\ 0.0000481,\rho_{y_{1}x_{1}}=0.337,\rho_{y_{2}x_{2}}=0.496,T_{M_{1}}=61.623,T_{M_{2}}=45.287,DM_{1}=61.600,\\ DM_{2}=45.242,Sk_{1}=0.300,Sk_{2}=0.500,QA_{1}=54.287,QA_{2}=48.940,QD_{1}=6.840,\\ QD_{2}=8.614,MAD_{1}=8.100,MAD_{2}=10.200,QR_{1}=13.721,QR_{2}=17.201,\\ MR_{1}=61.500,MR_{2}=45. \end{array}$$

### 6.3. Discussion and Results

Newly introduced transformation-based estimators have great benefits when compared with conventional median estimation methods. These estimators always had smaller mean squared errors in both simulated and real data which implies greater accuracy in determining the population median. These proved to be reliable with symmetric, moderately skewed, and heavy-tailed data, and the performance was not dependent on the characteristics of the distribution. Table 4, Table 5, Table 6 and Table 7 summarize the main results and demonstrate different kinds of significant trends.

**Figure 1:**
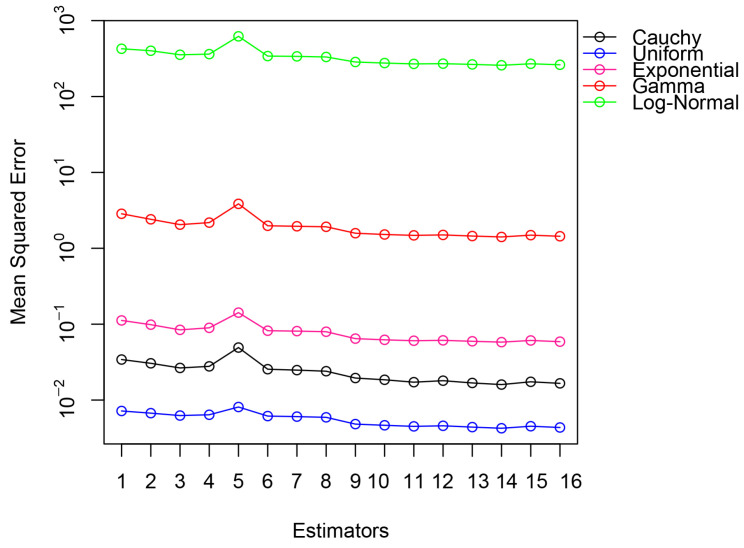
Mean squared error (MSE) values for the proposed and conventional estimators are calculated from simulated datasets and depicted in the graphical summary.

**Figure 2:**
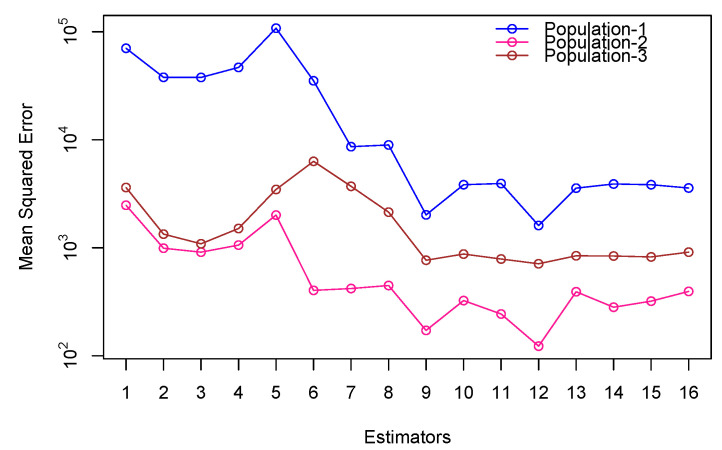
Mean squared error (MSE) values for the proposed and conventional estimators are calculated from real three populations and depicted in the graphical summary.

**Figure 3:**
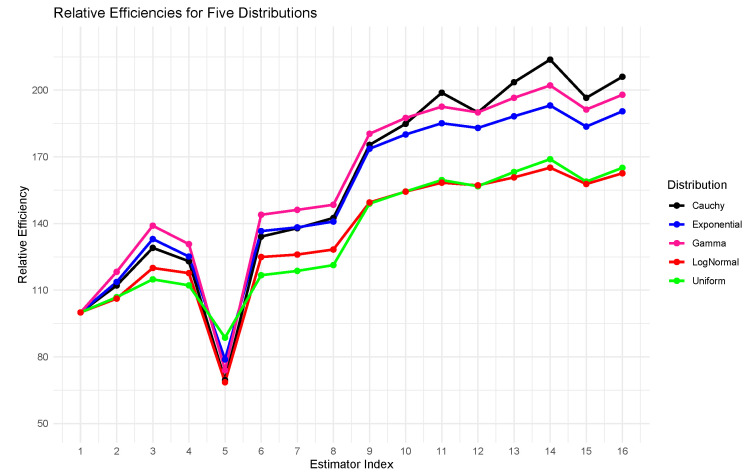
Relative efficiency values for the proposed and conventional estimators are calculated from simulated datasets and depicted in the graphical summary.

**Figure 4:**
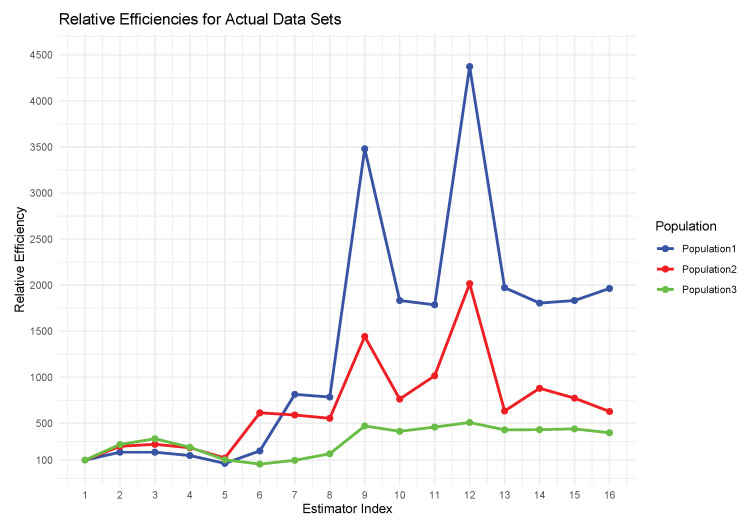
Felative efficiency values for the proposed and conventional estimators are calculated from real three populations and depicted in the graphical summary.

- The proposed transformation-based estimators continued to outperform the existing ones with both simulated and real-world data. They obtained significantly smaller errors in the mean squared in the simulated populations (Table 4, Figure 1), which suggests a higher accuracy of the median estimation. The same trends were noted with empirical data; the approach was highly effective (Table 5, Figure 2). Regardless of the underlying data, the suggested estimators performed well and effectively, achieving the smallest values of MSE in any practical situation where they could be applied. Such findings show that the transformation-based method is useful in the production of reliable and accurate median estimates in a variety of contexts.
- Figure 1 and Figure 2 reveal that the proposed estimators perform reliably across varying correlation levels between study and auxiliary variables. As indicated in Table 4 and Table 5, their efficiency is sustained even when $n_{h}$ is considerably smaller than $m_{h}$, making them suitable for cost-limited stratified sampling.
- In addition to Figure 1 and Figure 2, the comparative patterns shown in Figure 3 and Figure 4 clearly demonstrate the consistency of the proposed estimators across both simulated and real data settings. Figure 3 highlights the relative efficiency trends presented in Table 6, where the quantile based estimators such as ${\hat{E}}_{5st}$, ${\hat{E}}_{6st}$, and ${\hat{E}}_{8st}$ show much higher efficiency values compared with traditional approaches, regardless of the shape of the distribution or the degree of skewness. The efficiency curves remain stable and clearly separated from the existing methods, confirming the strength of the transformation based formulation under a wide range of population conditions. In a similar way, Figure 4, which summarizes the empirical findings from Table 7, supports this pattern using real data. The proposed estimators provide higher precision and smaller sampling variability in all three populations, showing that the quantile transformations work effectively beyond the simulated framework. Taken together, the graphical and tabular evidence confirms that the new family of estimators improves numerical accuracy and gives a more reliable performance in different and realistic data situations.
- When the underlying population deviates from normality, especially when there is moderate to high skewness or a small percentage of extreme observations in the data, the suggested estimators show notable efficiency gains. According to simulation results, the quantile-based transformations maintain stability and produce smaller mean squared errors than conventional ratio or regression-type estimators when applied to right-skewed and heavy-tailed distributions, such as exponential or log-normal models. The performance advantage increases further when contamination levels of up to 10–15 % are added, demonstrating the suggested class of estimators stability in irregular and heterogeneous data environments.

**Table 4:** MSE values under stratified two-phase sampling for simulation data sets.

Estimator	C(21,16)	Uni(17,24)	Exp(0.5)	Gam(23,15)	LN(11,6)
${\hat{M}}_{yst}$	$3.42\times 10^{-2}$	$7.18\times 10^{-3}$	$1.12\times 10^{-1}$	$2.85\times 10^{0}$	$4.26\times 10^{2}$
${\hat{M}}_{A_{st}}$	$3.05\times 10^{-2}$	$6.72\times 10^{-3}$	$9.85\times 10^{-2}$	$2.41\times 10^{0}$	$4.01\times 10^{2}$
${\hat{M}}_{D_{1st}}$	$2.65\times 10^{-2}$	$6.25\times 10^{-3}$	$8.42\times 10^{-2}$	$2.05\times 10^{0}$	$3.55\times 10^{2}$
${\hat{M}}_{Re_{st}}$	$2.78\times 10^{-2}$	$6.40\times 10^{-3}$	$8.95\times 10^{-2}$	$2.18\times 10^{0}$	$3.62\times 10^{2}$
${\hat{M}}_{Pe_{st}}$	$4.92\times 10^{-2}$	$8.10\times 10^{-3}$	$1.42\times 10^{-1}$	$3.85\times 10^{0}$	$6.21\times 10^{2}$
${\hat{M}}_{D_{2st}}$	$2.55\times 10^{-2}$	$6.15\times 10^{-3}$	$8.20\times 10^{-2}$	$1.98\times 10^{0}$	$3.41\times 10^{2}$
${\hat{M}}_{D_{3st}}$	$2.48\times 10^{-2}$	$6.05\times 10^{-3}$	$8.10\times 10^{-2}$	$1.95\times 10^{0}$	$3.38\times 10^{2}$
${\hat{M}}_{D_{4st}}$	$2.40\times 10^{-2}$	$5.92\times 10^{-3}$	$7.95\times 10^{-2}$	$1.92\times 10^{0}$	$3.32\times 10^{2}$
${\hat{E}}_{1_{st}}$	$1.95\times 10^{-2}$	$4.82\times 10^{-3}$	$6.45\times 10^{-2}$	$1.58\times 10^{0}$	$2.85\times 10^{2}$
${\hat{E}}_{2_{st}}$	$1.85\times 10^{-2}$	$4.65\times 10^{-3}$	$6.22\times 10^{-2}$	$1.52\times 10^{0}$	$2.76\times 10^{2}$
${\hat{E}}_{3_{st}}$	$1.72\times 10^{-2}$	$4.50\times 10^{-3}$	$6.05\times 10^{-2}$	$1.48\times 10^{0}$	$2.69\times 10^{2}$
${\hat{E}}_{4_{st}}$	$1.80\times 10^{-2}$	$4.58\times 10^{-3}$	$6.12\times 10^{-2}$	$1.50\times 10^{0}$	$2.71\times 10^{2}$
${\hat{E}}_{5_{st}}$	$1.68\times 10^{-2}$	$4.40\times 10^{-3}$	$5.95\times 10^{-2}$	$1.45\times 10^{0}$	$2.65\times 10^{2}$
${\hat{E}}_{6_{st}}$	$1.60\times 10^{-2}$	$4.25\times 10^{-3}$	$5.80\times 10^{-2}$	$1.41\times 10^{0}$	$2.58\times 10^{2}$
${\hat{E}}_{7_{st}}$	$1.74\times 10^{-2}$	$4.52\times 10^{-3}$	$6.10\times 10^{-2}$	$1.49\times 10^{0}$	$2.70\times 10^{2}$
${\hat{E}}_{8_{st}}$	$1.66\times 10^{-2}$	$4.35\times 10^{-3}$	$5.88\times 10^{-2}$	$1.44\times 10^{0}$	$2.62\times 10^{2}$

**Table 5:** Analysis of mean squared error performance using empirical populations.

Estimator	Population-1	Population-2	Population-3
${\hat{M}}_{yst}$	$7.04\times 10^{4}$	$2.48\times 10^{3}$	$3.62\times 10^{3}$
${\hat{M}}_{Ast}$	$3.79\times 10^{4}$	$9.92\times 10^{2}$	$1.34\times 10^{3}$
${\hat{M}}_{D_{1st}}$	$3.78\times 10^{4}$	$9.12\times 10^{2}$	$1.09\times 10^{3}$
${\hat{M}}_{Re_{st}}$	$4.68\times 10^{4}$	$1.06\times 10^{3}$	$1.51\times 10^{3}$
${\hat{M}}_{Pe_{st}}$	$1.08\times 10^{5}$	$2.01\times 10^{3}$	$3.47\times 10^{3}$
${\hat{M}}_{D_{2st}}$	$3.52\times 10^{4}$	$4.04\times 10^{2}$	$6.32\times 10^{3}$
${\hat{M}}_{D_{3st}}$	$8.64\times 10^{3}$	$4.20\times 10^{2}$	$3.71\times 10^{3}$
${\hat{M}}_{D_{4st}}$	$8.96\times 10^{3}$	$4.48\times 10^{2}$	$2.14\times 10^{3}$
${\hat{E}}_{1_{st}}$	$2.02\times 10^{3}$	$1.72\times 10^{2}$	$7.68\times 10^{2}$
${\hat{E}}_{2_{st}}$	$3.84\times 10^{3}$	$3.25\times 10^{2}$	$8.76\times 10^{2}$
${\hat{E}}_{3_{st}}$	$3.94\times 10^{3}$	$2.44\times 10^{2}$	$7.88\times 10^{2}$
${\hat{E}}_{4_{st}}$	$1.61\times 10^{3}$	$1.23\times 10^{2}$	$7.12\times 10^{2}$
${\hat{E}}_{5_{st}}$	$3.57\times 10^{3}$	$3.92\times 10^{2}$	$8.44\times 10^{2}$
${\hat{E}}_{6_{st}}$	$3.90\times 10^{3}$	$2.82\times 10^{2}$	$8.40\times 10^{2}$
${\hat{E}}_{7_{st}}$	$3.84\times 10^{3}$	$3.21\times 10^{2}$	$8.24\times 10^{2}$
${\hat{E}}_{8_{st}}$	$3.58\times 10^{3}$	$3.95\times 10^{2}$	$9.12\times 10^{2}$

**Table 6:** Relative efficiencies (baseline = ${\hat{M}}_{yst}$) for simulation data sets.

Estimator	C(21,16)	Uni(17,24)	Exp(0.5)	Gam(23,15)	LN(11,6)
${\hat{M}}_{yst}$	100.00	100.00	100.00	100.00	100.00
${\hat{M}}_{A_{st}}$	112.13	106.85	113.71	118.26	106.23
${\hat{M}}_{D_{1st}}$	129.06	114.88	133.02	139.02	120.00
${\hat{M}}_{Re_{st}}$	123.02	112.19	125.14	130.73	117.68
${\hat{M}}_{Pe_{st}}$	69.51	88.64	78.87	74.03	68.60
${\hat{M}}_{D_{2st}}$	134.12	116.75	136.59	143.94	124.93
${\hat{M}}_{D_{3st}}$	137.90	118.68	138.27	146.15	126.04
${\hat{M}}_{D_{4st}}$	142.50	121.28	140.88	148.44	128.31
${\hat{E}}_{1_{st}}$	175.38	148.96	173.64	180.38	149.47
${\hat{E}}_{2_{st}}$	184.86	154.41	180.06	187.50	154.35
${\hat{E}}_{3_{st}}$	198.84	159.56	185.12	192.57	158.36
${\hat{E}}_{4_{st}}$	190.00	156.77	183.01	190.00	157.20
${\hat{E}}_{5_{st}}$	203.57	163.18	188.24	196.55	160.75
${\hat{E}}_{6_{st}}$	213.75	168.94	193.10	202.13	165.12
${\hat{E}}_{7_{st}}$	196.55	158.85	183.61	191.28	157.78
${\hat{E}}_{8_{st}}$	206.02	165.06	190.48	197.92	162.60

**Table 7:** Relative efficiencies (baseline = ${\hat{M}}_{yst}$) for actual data sets.

Estimator	Population-1	Population-2	Population-3
${\hat{M}}_{yst}$	100	100	100
${\hat{M}}_{Ast}$	186	250	270
${\hat{M}}_{D_{1st}}$	186	272	332
${\hat{M}}_{Re_{st}}$	150	234	240
${\hat{M}}_{Pe_{st}}$	65	123	104
${\hat{M}}_{D_{2st}}$	200	614	57
${\hat{M}}_{D_{3st}}$	814	590	98
${\hat{M}}_{D_{4st}}$	785	554	169
${\hat{E}}_{1st}$	**3480**	**1442**	**471**
${\hat{E}}_{2st}$	**1833**	**763**	**413**
${\hat{E}}_{3st}$	**1786**	**1016**	**459**
${\hat{E}}_{4st}$	**4373**	**2016**	**509**
${\hat{E}}_{5st}$	**1972**	**633**	**429**
${\hat{E}}_{6st}$	**1805**	**880**	**431**
${\hat{E}}_{7st}$	**1833**	**773**	**439**
${\hat{E}}_{8st}$	**1964**	**628**	**397**

## 7. Conclusions

This study developed a family of transformation-based median estimators for stratified two-phase sampling using quantile and robust-scale measures. The transformations considered include quartile deviation, interquartile range, median absolute deviation, trimmed mean, decile mean, quartile average, mid-range, and skewness. These measures provide a balanced way to use auxiliary information while maintaining resistance to outliers and skewness. Results from simulation and empirical analyses consistently demonstrated that the new estimators achieve lower mean squared errors and higher relative efficiencies than conventional median estimators. These improvements hold across various population structures, confirming the robustness and adaptability of the proposed approach.

Most existing median estimators in stratified two-phase sampling assume symmetric distributions and stable auxiliary information between phases. Their performance declines with skewed populations, outliers, or heterogeneous strata. Traditional ratio, regression, and exponential estimators were designed for standard conditions and cannot adapt to extreme data or varied distributions. This study proposed a generalized double-exponential median estimator using quantile-based transformations that capture spread, skewness, and tail behavior, making it more efficient and stable in irregular and non-normal stratified populations.

The proposed framework is highly flexible across survey types, samples, and strata, making it useful for socio-economic, environmental, and health studies. Future work may extend it to multi-phase and multi-auxiliary settings, complex sampling designs (clustered, multistage, or adaptive), and robust location measures such as quantile regression or trimmed means. Further research can also develop optimal data-based calibration rules and confidence interval procedures to enhance efficiency and applicability in modern, high-dimensional survey contexts. 

## Figures and Tables

**Table 1:** List of notations used in the study.

Symbol	Description	Symbol	Description
*N*	Population size	*L*	Number of strata
$N_{h}$	Units in stratum *h*	$m_{h}$	First-phase sample size in stratum *h*
$n_{h}$	Second-phase sample size in stratum *h*	*m*	Total first-phase sample size
*n*	Total second-phase sample size	*Y*	Study variable
*X*	Auxiliary variable	$M_{yh},M_{xh}$	Population medians of *Y* and *X*
${\hat{M}}_{yh},{\hat{M}}_{xh}$	Second-phase sample medians	${\dot{M}}_{xh}$	First-phase sample median of *X*
$f_{yh},f_{xh}$	Probability density at medians	$W_{h}$	Stratum weight
$\rho_{yxh}$	Correlation between *Y* and *X*	$P_{11h}$	Joint probability function
$e_{0h},e_{1h},e_{2h}$	Relative error terms	$C_{M_{yh}},C_{M_{xh}}$	Median coefficients
$C_{M_{yxh}}$	Covariance coefficient	$\theta_{1h},\theta_{2h},\theta_{3h}$	Sampling constants
$Q_{1h},Q_{3h}$	First and third quartiles	$IQR_{h}$	Interquartile range
$QD_{h}$	Quartile deviation	$QA_{h}$	Quartile average
$TM_{h}$	Trimmed mean	$DM_{h}$	Decile mean
$MAD_{h}$	Median absolute deviation	$MR_{h}$	Mid-range of $X_{h}$
$\sigma_{X_{h}}$	Standard deviation of *X*	$Sk(X_{h})$	Skewness of *X*
$E_{1st}$–$E_{8st}$	Proposed estimators (quantile-based)	MSE	Mean squared error
Bias	Bias of estimator	$k_{1h},k_{2h}$	Transformation constants
$t_{ih},V_{ih}$	Calibration parameters	$\mathrm{Cov}(M_{y_{h}},M_{x_{h}})$	Covariance of *Y* and *X* in *h* stratum

**Table 2:** Proposed estimator transformations in stratified two-phase sampling.

Estimator	$t_{1h}$	$t_{2h}$	$t_{3h}$	$t_{4h}$
${\hat{E}}_{st_{1}}$	$QD_{h}$	$MAD_{h}$	1	$X_{h\max}-X_{h\min}$
${\hat{E}}_{st_{2}}$	$TM_{h}$	$MR_{h}$	1	$IQR_{h}$
${\hat{E}}_{st_{3}}$	$DM_{h}$	$MAD_{h}$	1	$QD_{h}$
${\hat{E}}_{st_{4}}$	$\mathrm{Skewness}(X_{h})$	1	1	$Q_{3h}-Q_{2h}$
${\hat{E}}_{st_{5}}$	$\log(Q_{3h}+1)$	$\log(Q_{1h}+1)$	1	$\log(MR_{h}+1)$
${\hat{E}}_{st_{6}}$	$QA_{h}$	$\sigma_{X_{h}}$	1	$QD_{h}$
${\hat{E}}_{st_{7}}$	$X_{\mathrm{median}}$	$IQR_{h}$	1	$MAD_{h}$
${\hat{E}}_{st_{8}}$	$\sqrt{Q_{1h}\cdot Q_{3h}}$	$\sqrt{X_{h\max}\cdot X_{h\min}}$	1	$\sqrt{IQR_{h}}$

## Data Availability

The original contributions presented in this study are included in the article. Further inquiries can be directed to the corresponding author.
